# Supplementary material for: Mitochondrial stress-activated cGAS-STING pathway inhibits thermogenic program and contributes to overnutrition-induced obesity in mice
Source: Commun Biol. 2020 May 22;3:257. doi: 10.1038/s42003-020-0986-1 (PMC7244732; doi:10.1038/s42003-020-0986-1)
Supplement: Supplementary file 1 — Supplementary Information [file 42003_2020_986_MOESM1_ESM.pdf]

Supplementary Fig. 1.

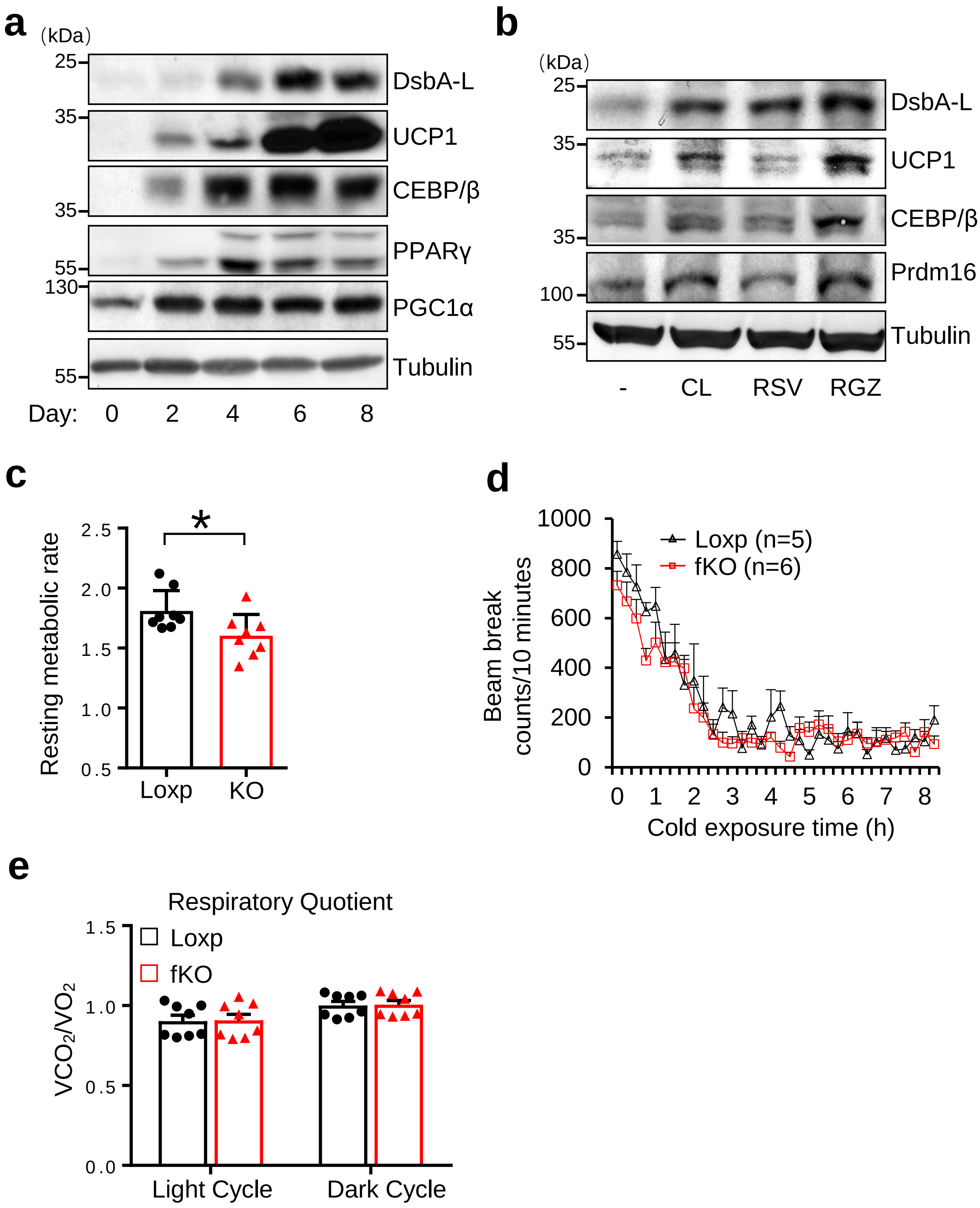

Supplementary Figure 1. DsbA-L is positively correlated with thermogenic gene expression and resting metabolic rate.

a Immunoblot analysis of DsbA-L, UCP1, C/EBPβ, PPARγ and PGC1α expression during brown adipocyte differentiation. **b** Immunoblot analysis of DsbA-L, UCP1, C/EBPβ and Prdm16 expression in brown adipocytes treated with CL316243, resveratrol or rosiglitazone for 16 hr. **c** Resting metabolic rate of DsbA-L^{fKO} and loxp control mice. **d** The activities of DsbA-L^{fKO} and loxp control mice (n=8 for each group) exposed to cold (4°C) under the feeding conditions at different time points as indicated. **e** The respiratory quotient of DsbA-L^{fKO} and loxp control mice (n=8 for each group) was measured during a 48 hr period, including two light/dark cycles. Data are presented as mean ± SEM of biologically independent samples, *$p < 0.05$ by unpaired two-tailed t-test.

Supplementary Fig. 2.

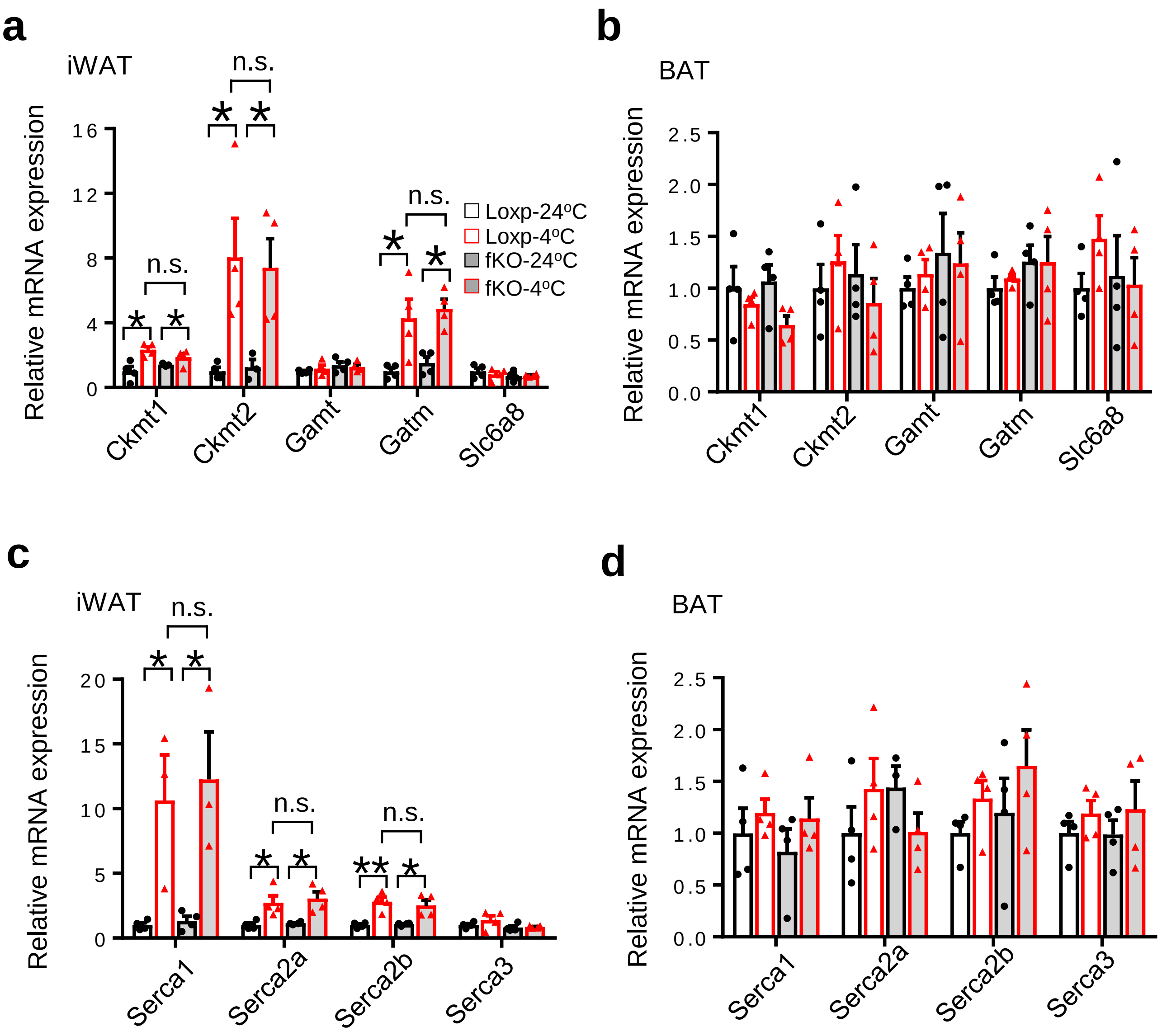

Supplementary Figure 2. Fat-specific knockout of DsbA-L has no effect on creatine and calcium cycle-related gene expression in mice.

a-b The mRNA levels of creatine-metabolism-related genes in (**a**) iWAT and (**b**) BAT of DsbA-L^{fKO} and loxp control mice (n=4 for each group) were determined by qPCR and normalized to β-actin. **c-d** The mRNA levels of calcium cycle-related genes in (**c**) iWAT and (**d**) BAT of DsbA-L^{fKO} and loxp control mice (n=8 for each group) were determined by qPCR and normalized to β-actin. Data are presented as mean ± SEM of biologically independent samples, *$p < 0.05$ and **$p < 0.01$ by one-way ANOVA.

Supplementary Fig. 3.

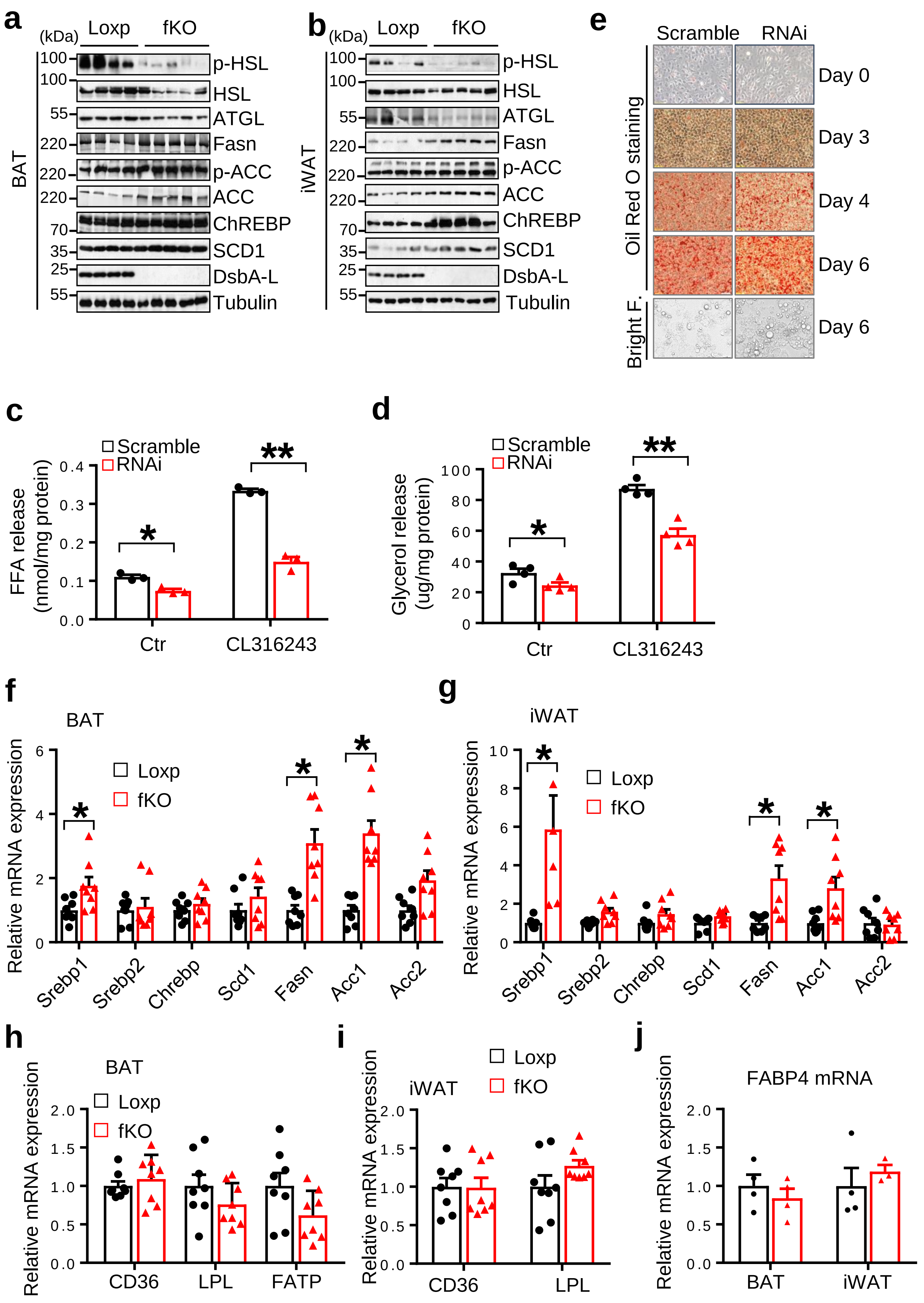

Supplementary Figure 3. Fat-specific knockout of DsbA-L impairs lipid metabolism.

a-b Immunoblot analysis of the expression of lipid metabolism-related genes such as ATGL, FASN, ChREBP, SCD1 and the phosphorylation of ACC and HSL in (**a**) BAT and (**b**) iWAT of DsbA-L^{fKO} (n=4) and loxp control (n=5) mice. **c** Free fatty acid and **d** glycerol release from DsbA-L-suppressed brown adipocytes treated with or without 1μM CL316243 (n=3 for each group) for 16 hr. **e** Representative Oil Red O stain or bright field photo during DsbA-L-suppressed brown adipocytes or scramble control cells differentiation. **f-g** The mRNA levels of lipid synthesis genes in (**f**) BAT and (**g**) iWAT of DsbA-L^{fKO} and loxp control mice (n=8 for each group). **h-i** The mRNA levels of lipid uptake-related genes in (**h**) BAT and (**i**) iWAT of DsbA-L^{fKO} and loxp control mice (n=8 for each group) were determined by qPCR and normalized to β-actin. **j** The mRNA levels of FABP4 in BAT and iWAT of DsbA-L^{fKO} and loxp control mice (n=4 for each group). Data are presented as mean ± SEM of biologically independent samples, *p < 0.05 and **p < 0.01 by unpaired two-tailed t-test.

Supplementary Fig. 4.

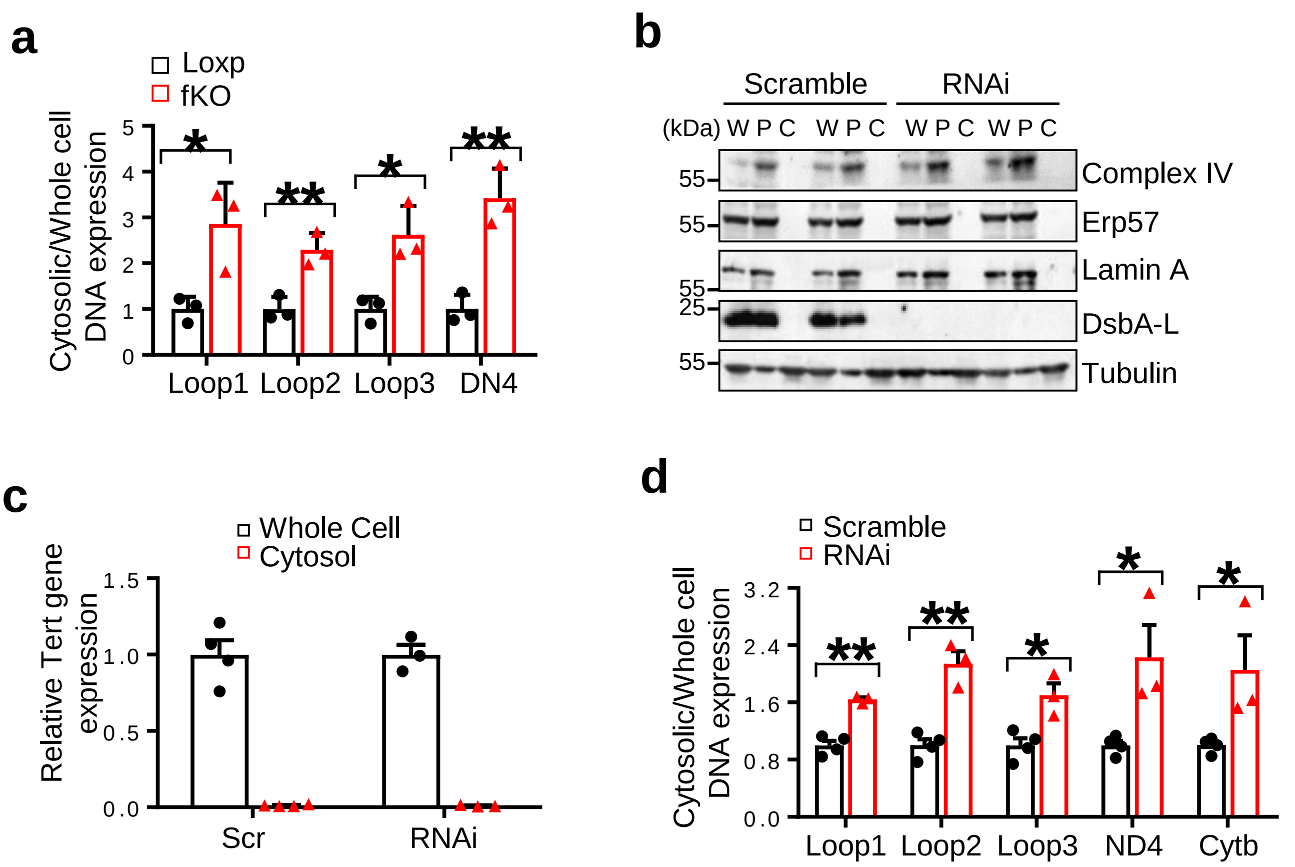

Supplementary Figure 4. DsbA-L deficiency results in increased mtDNA release into the cytosol in brown adipocytes.

a Cytosolic mtDNA content was quantitated via qPCR using mtDNA primers (Dloop1-3 and mtND4) in primary brown adipocytes from DsbA-L^{fKO} and loxp control mice (n=3 for each group). **b** Scramble and DsbA-L-suppressed brown adipocytes were subjected to digitonin fractionation. Whole-cell extracts (W), pellets (P) and cytosolic extracts (C) were immunoblotted using indicated antibodies. **c** The expression of nuclear-encoded Tert gene in whole-cell and cytosolic extract was measured from scramble (n=4) and DsbA-L-suppressed (n=3) brown adipocytes, indicating the high purity of cytosol fraction. **d** Cytosolic mtDNA content was quantitated via qPCR using mtDNA primers (Dloop1-3, mtND4 and Cytb) in brown scramble (n=4) and DsbA-L-suppressed (n=3) adipocytes. Data are presented as mean ± SEM of biologically independent samples, $*p < 0.05$ and $**p < 0.01$ by unpaired two-tailed t-test.

Supplementary Fig. 5.

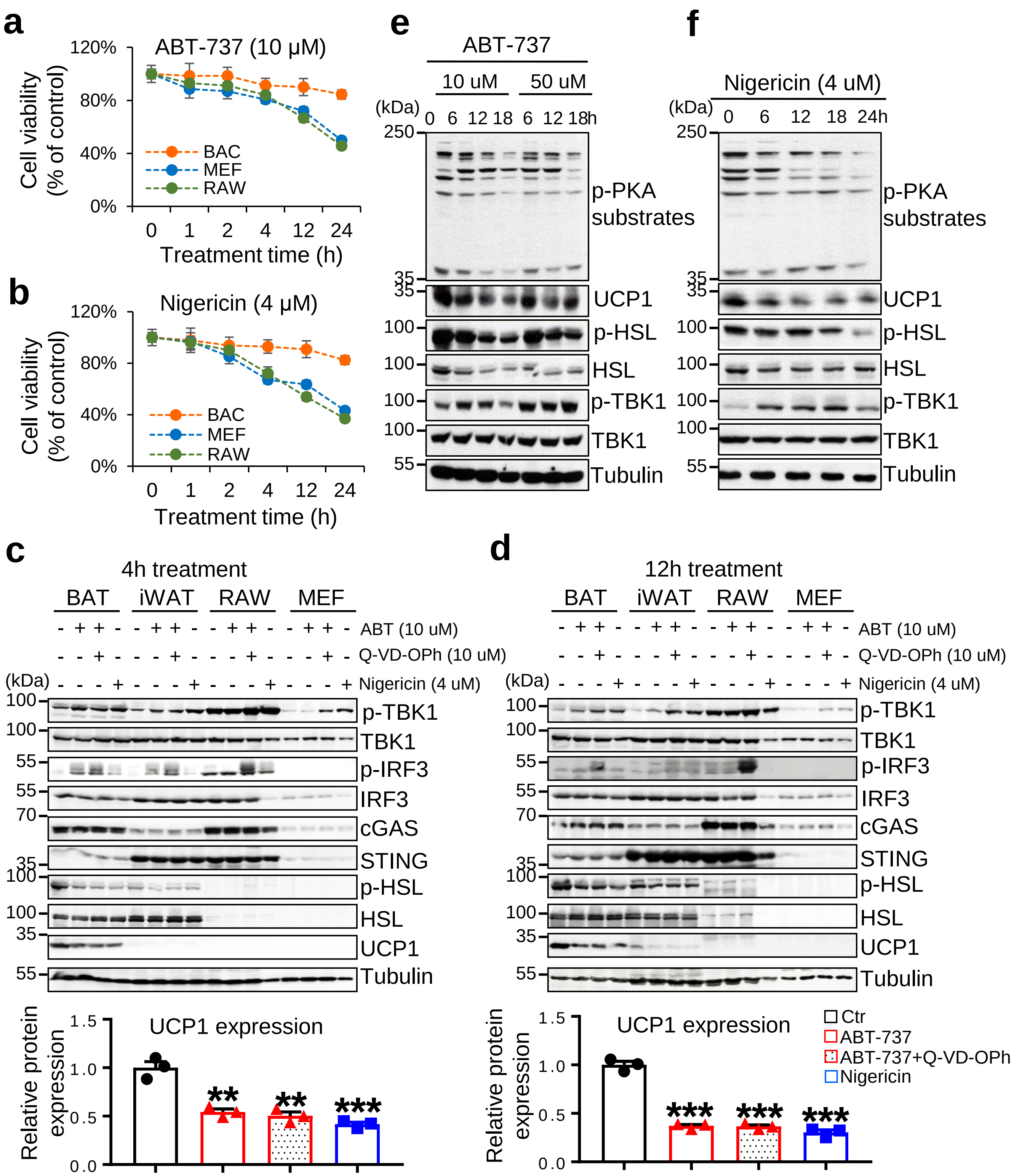

Supplementary Figure 5. mtDNA release-induced activation of the cGAS-STING pathway inhibits PKA signaling in brown adipocytes.

a-b MTT assay indicated cell viability of brown adipocytes (BAC), MEF and RAW264.7 cells treated with (**a**) 10 μM ABT-737 or (**b**) 4 μM nigericin at different time points as indicated. **c-d** Immunoblot analysis of the phosphorylation of TBK1, IRF3, HSL and UCP1, cGAS, STING expression in primary brown adipocytes, primary inguinal adipocytes, RAW264.7 and MEF cells treated with 4 μM nigericin or 10 μM ABT-737 in the presence or absence of Q-VD-OPh for (**c**) 4h or (**d**) 12 hr (n=3 for each group). The data was semi-quantified by Image J program. **e** Immunoblot analysis of UCP1 expression and the phosphorylation of PKA substrates, HSL, TBK1 and IRF3 in brown adipocytes treated with 10 μM or 50 μM ABT-737 at different time points as indicated. **f** Immunoblot analysis of UCP1 expression and the phosphorylation of PKA substrates, HSL, TBK1 and IRF3 in brown adipocytes treated with 4 μM nigericin at different time points as indicated.

Supplementary Fig. 6.

a

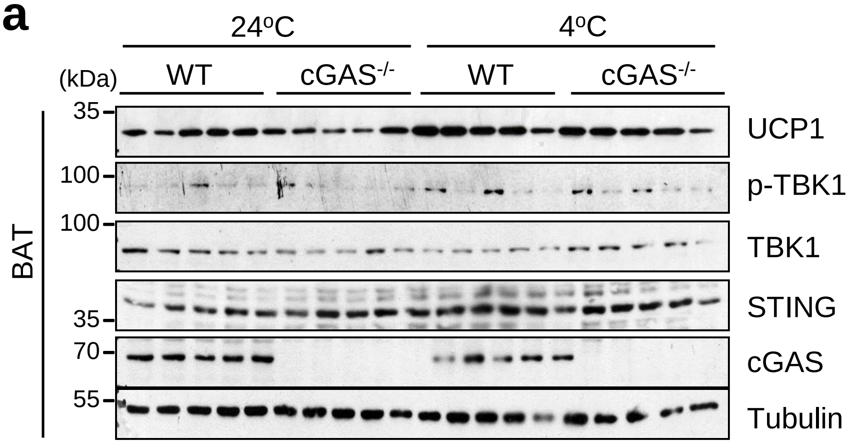

b

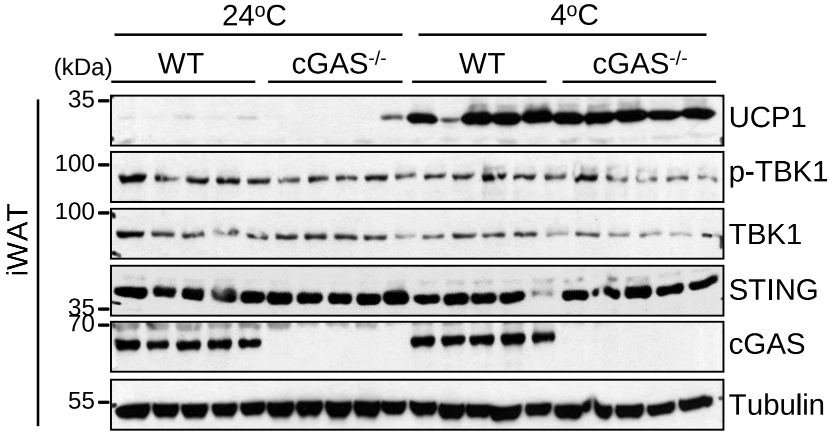

Supplementary Figure 6. Knockout of cGAS has no effect on UCP1 expression in mice. **a** Immunoblot analysis of UCP1, cGAS, STING expression and the phosphorylation of TBK1 in BAT from wild-type and cGAS knockout mice exposed to cold (4°C) or housed at room temperature (24°C) (n=5/group). **b** Immunoblot analysis of UCP1, cGAS, STING expression and the phosphorylation of TBK1 in iWAT from wild-type and cGAS knockout mice exposed to cold (4°C) or housed at room temperature (24°C) (n=5 for each group).

Fig. 1c

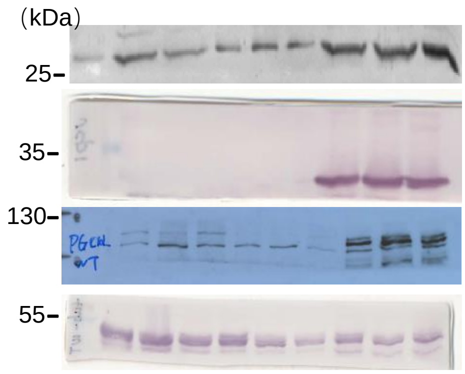

Fig. 2c

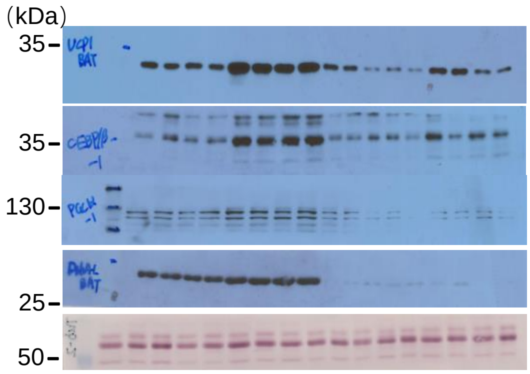

Fig. 2d

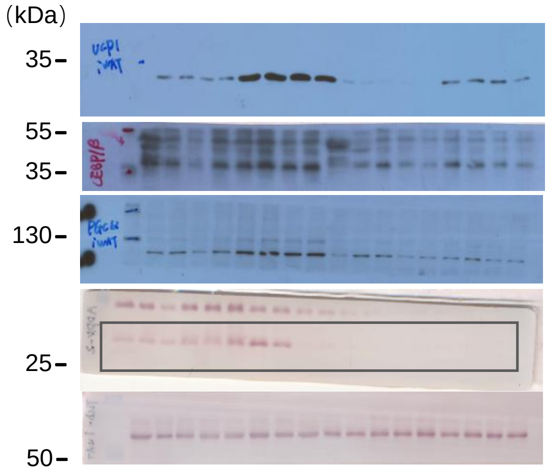

Fig. 2f

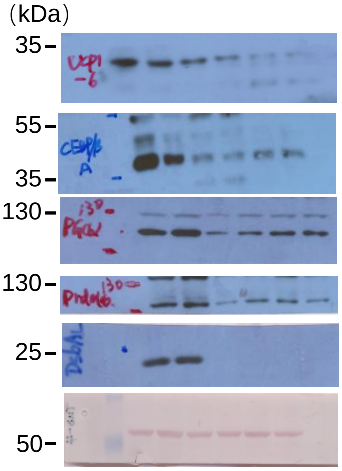

Fig. 2g

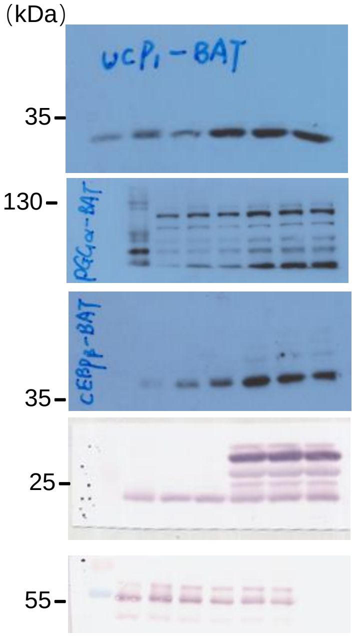

Fig. 2h

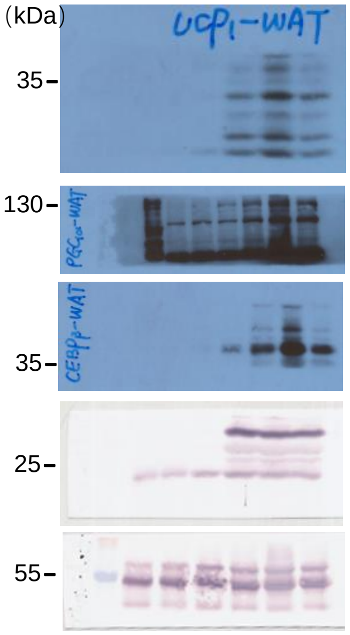

Fig. 4b

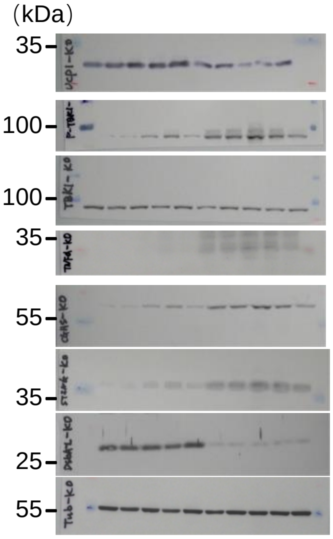

Fig. 4c

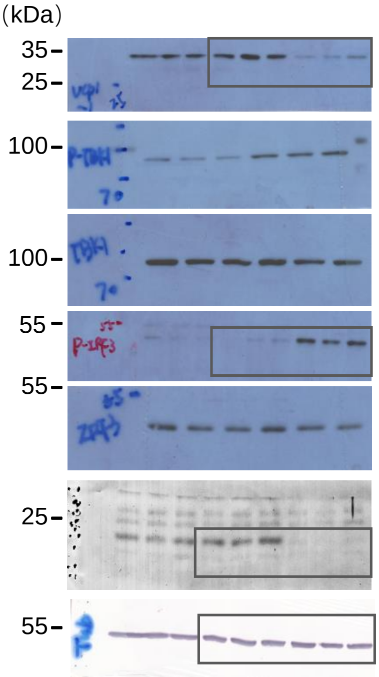

Fig. 4e

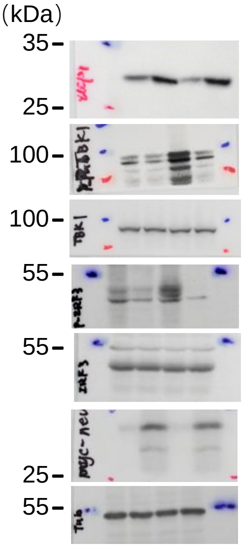

Fig. 4f & Fig. 4g

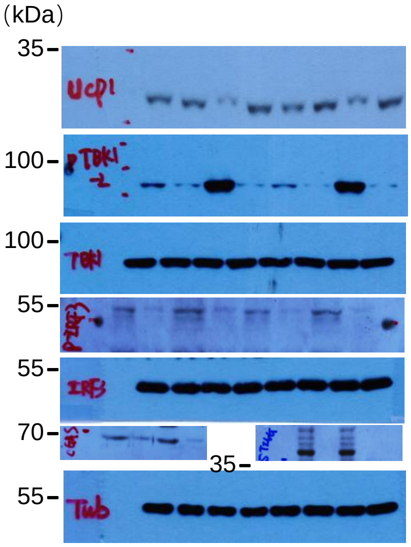

Fig. 4h

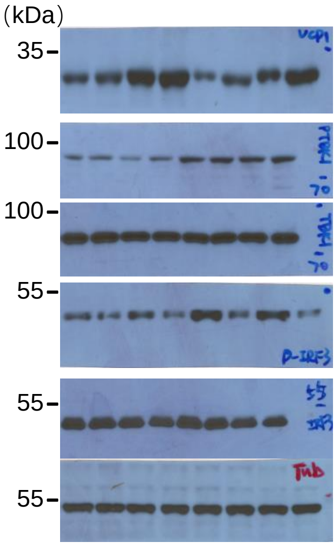

Fig. 4i

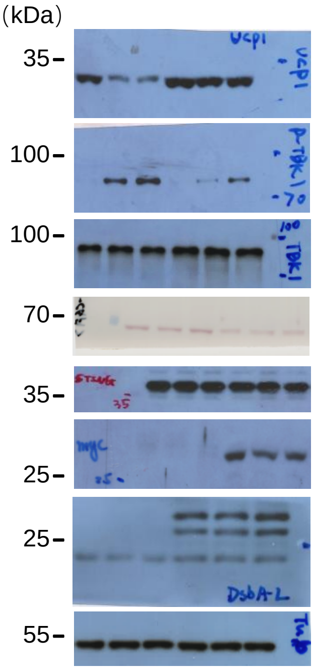

Fig. 5a

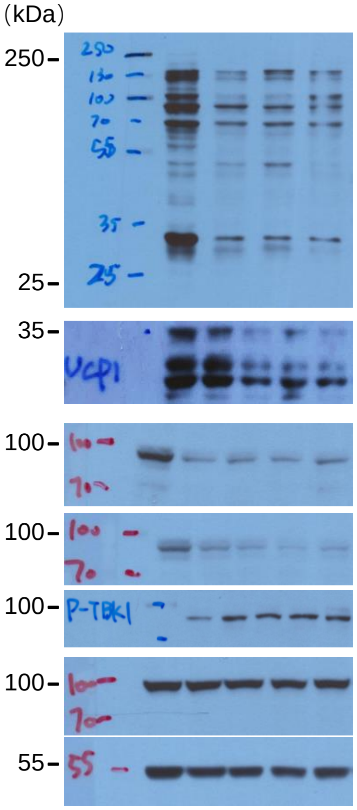

Fig. 5b

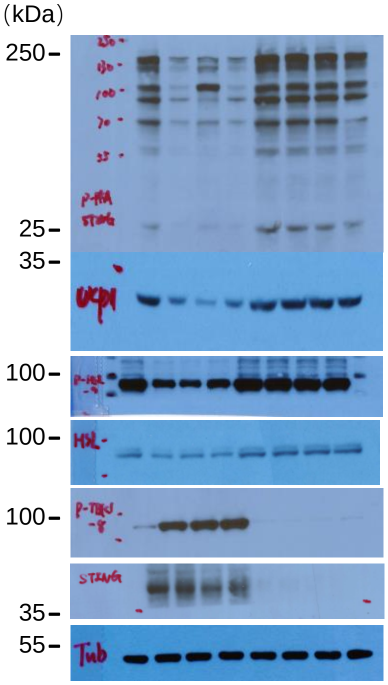

Fig. 5c

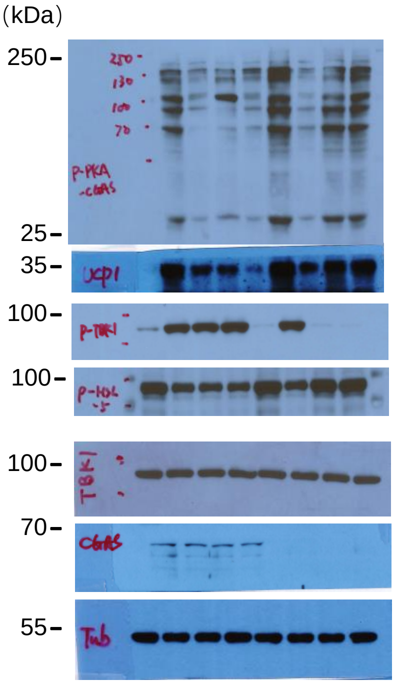

Fig. 5d & Fig. 5e

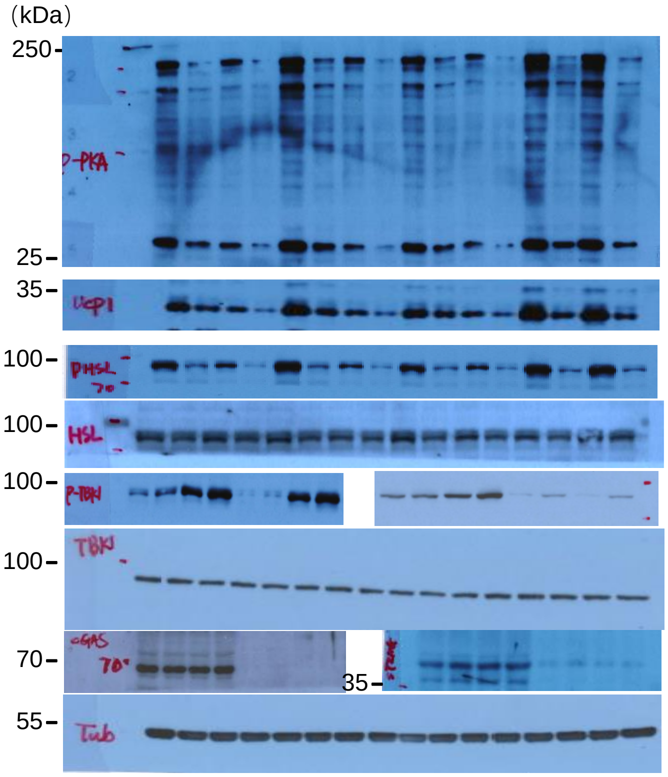

Fig. 5f

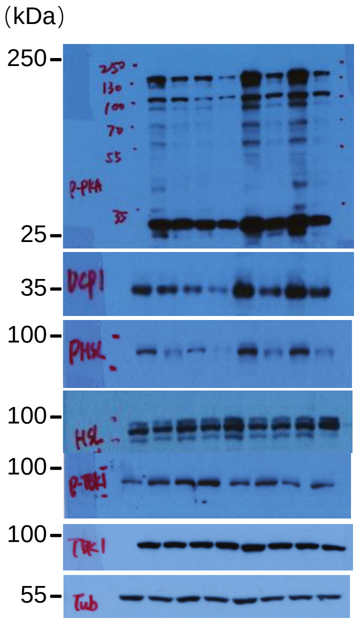

Fig. 6a

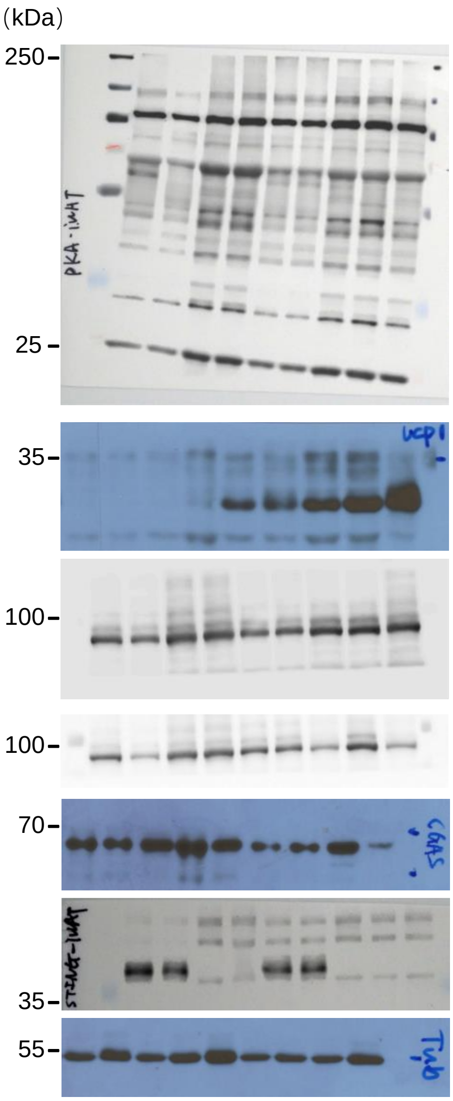
(kDa)
250
25
35
100
100
70
35
55
PKA-iWAT
UCP1
CGAS
Tub

Fig. 6b

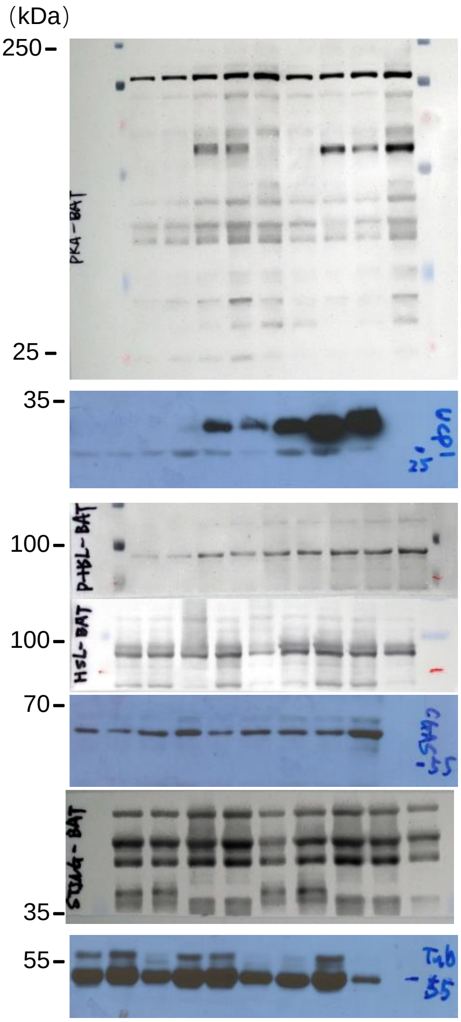
(kDa)
250
25
35
100
100
70
35
55
PKA-BAT
UCP1
P-HSL-BAT
HSL-BAT
CGAS
Tub

Fig. 6d

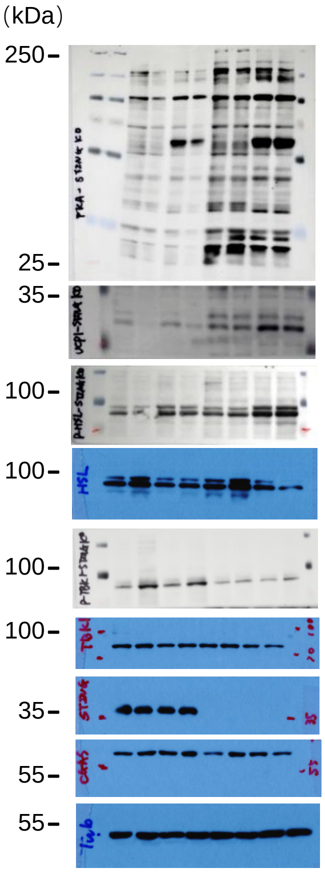

Fig. 6e

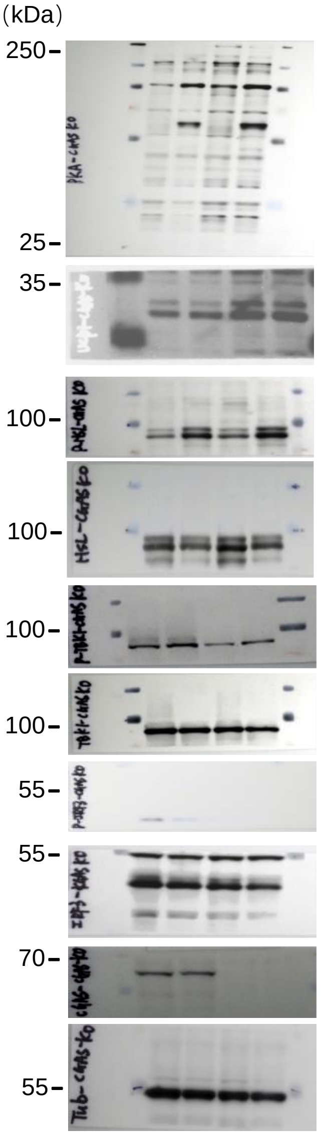

Fig. 6h

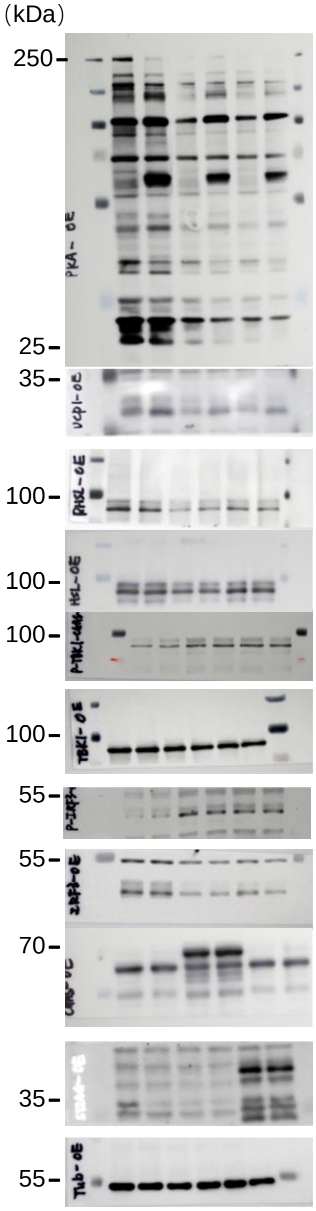

Suppl Fig.1a

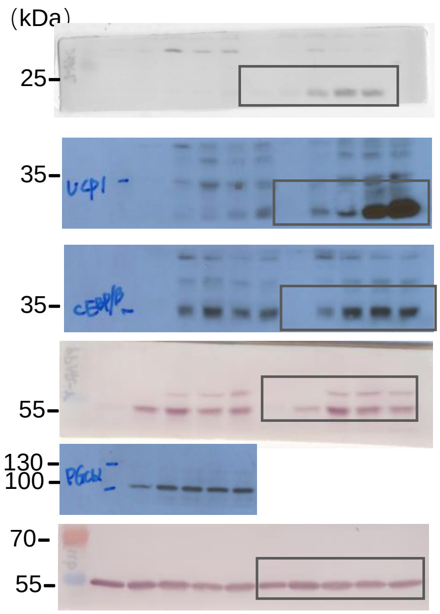

Suppl Fig.1b

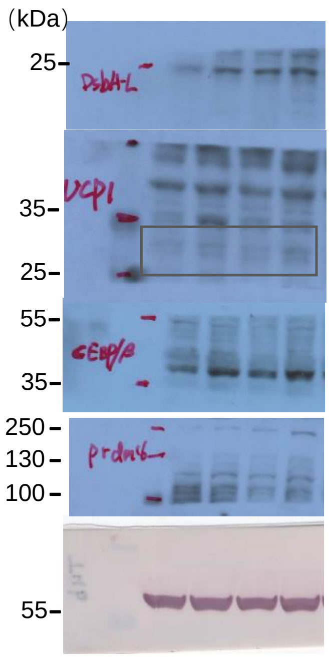

Suppl Fig.3a

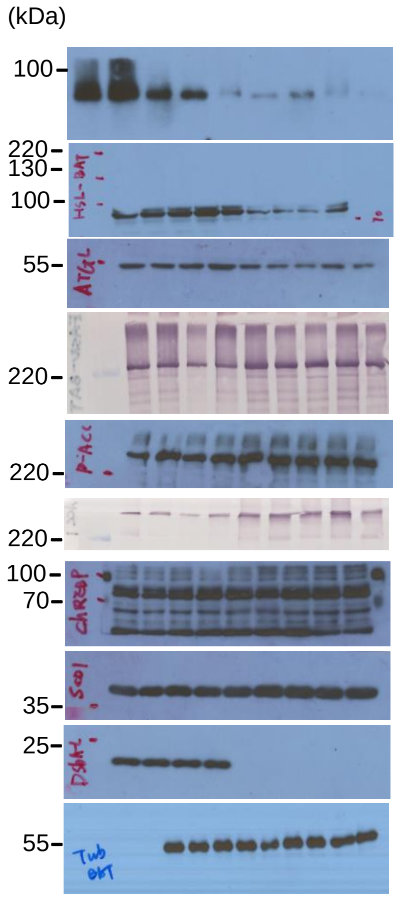

Suppl Fig.3b

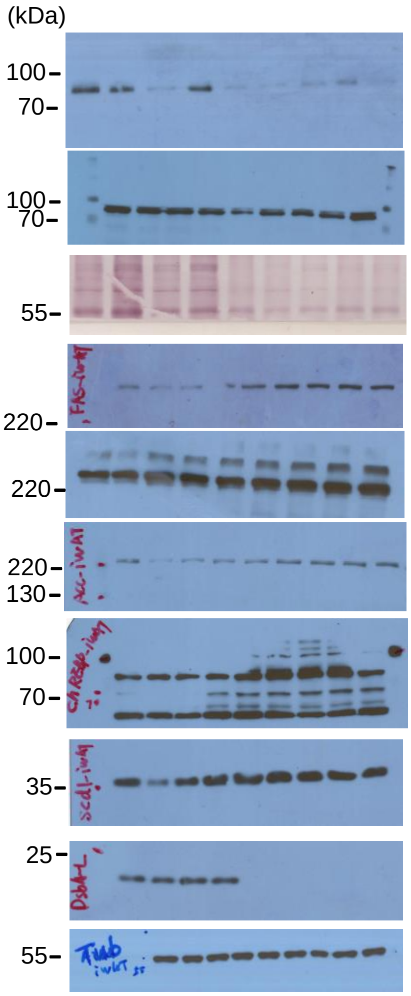

Suppl Fig. 4b

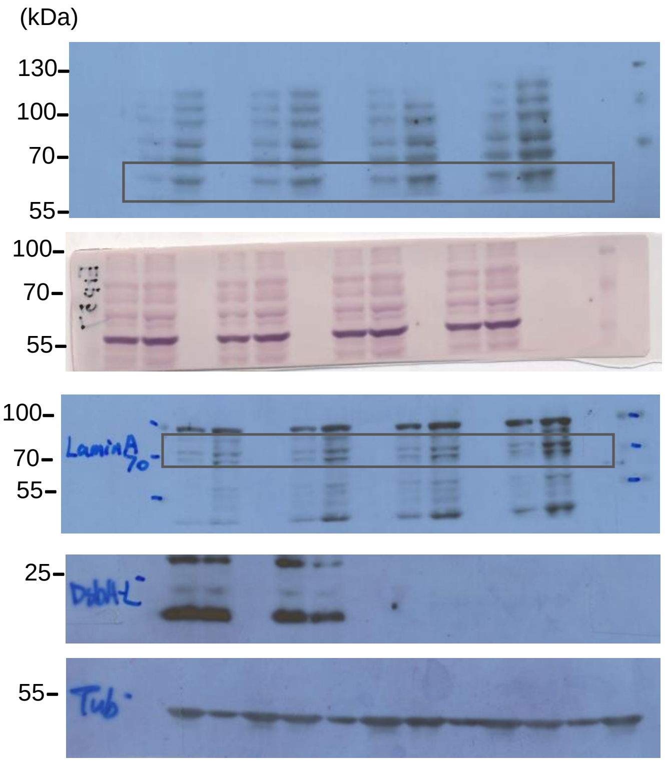

Suppl Fig. 5e

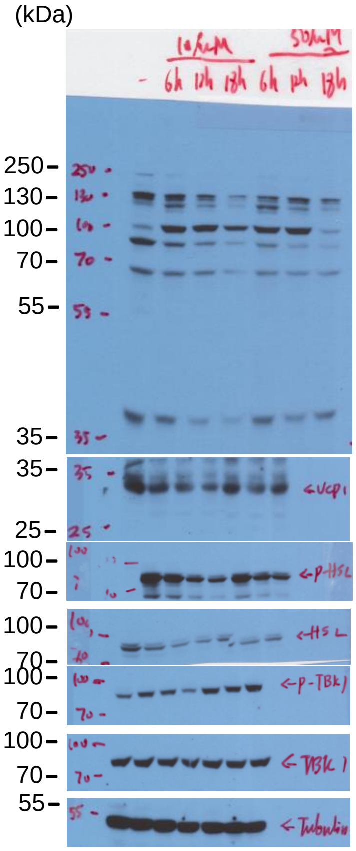

Suppl Fig. 5f

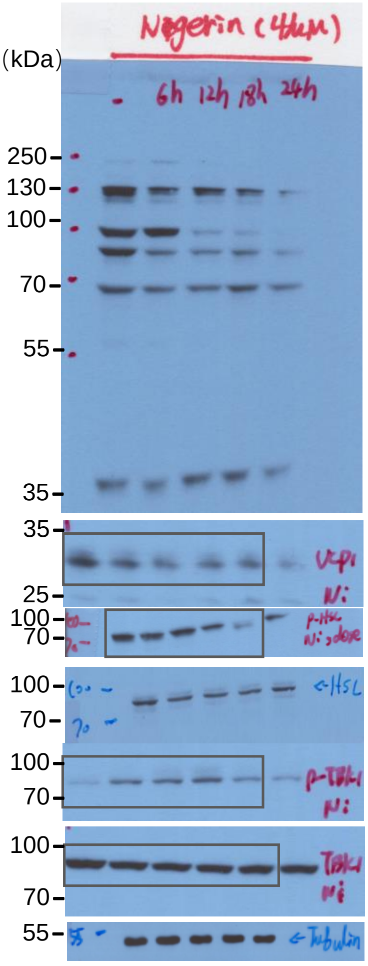

Suppl Fig. 5c

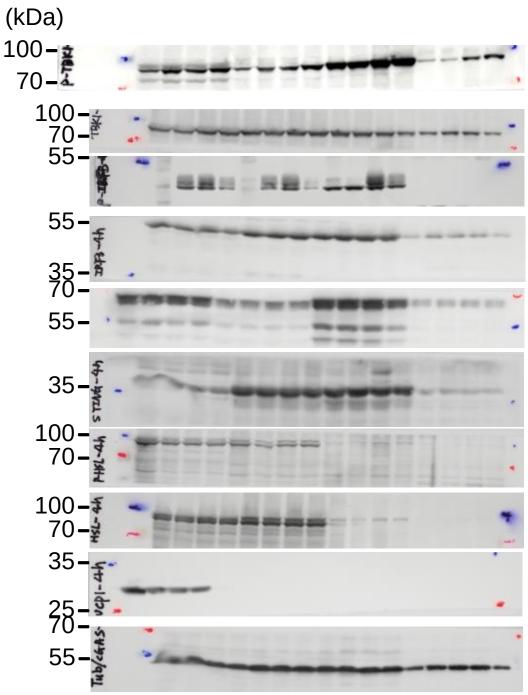

Suppl Fig. 5d

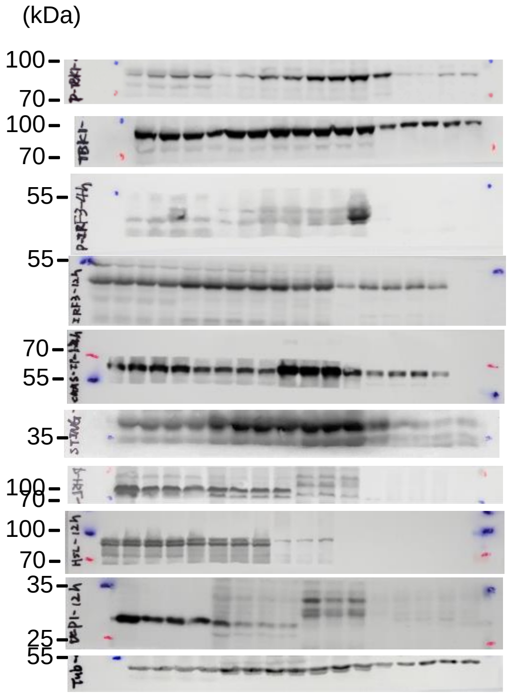

Suppl Fig. 6a

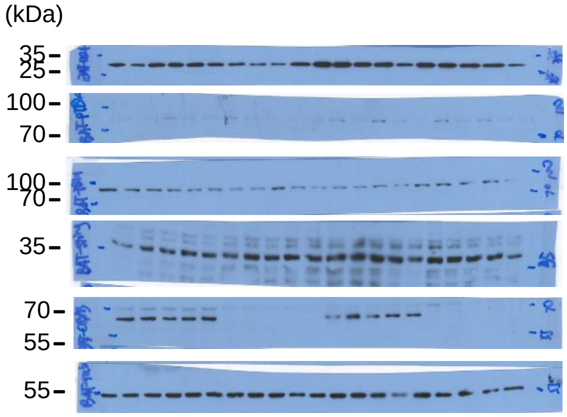

Suppl Fig. 6b

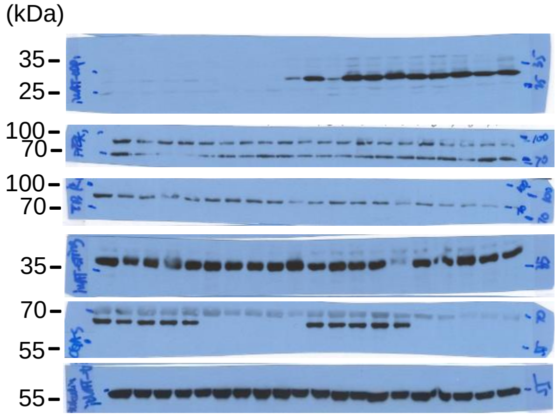

Supplementary Table 1: Primer pair sequences

Gene	Primer	Sequence	Gene	Primer	Sequence
DsbA-L	Forward	5’-ATGGATGCGTGTATGGTCTC-3’	SREBP1	Forward	5’-CCCTGTGTGTACTGGCCTTT-3’
	Reverse	5’-CAACAGTGGTGGGTAGCG-3’		Reverse	5’-TTGCGATGTCTCCAGAAGTG-3’
UCP1	Forward	5’-AAGACAGAAGAGCATAGCATTCAC-3’	SREBP2	Forward	5’-AAGCTGGGCGATGGATGAG-3’
	Reverse	5’-CCAGTCATACACTCCCACCTC-3’		Reverse	5’-ATCTCGTCGATGTCCCCG-3’
C/EBPβ	Forward	5’-TTCCTCTCCGACCTCTTC-3’	ChREBP	Forward	5’-CTGGGGACCTAAACAGGAGC-3’
	Reverse	5’-GCTCACGTAACCGTAGTC-3’		Reverse	5’-GAAGCCACCCTATAGCTCCC-3’
PGC1α	Forward	5’-CCGAAGACACTACAGGTTCCATAG-3’	SCD1	Forward	5’-CGCTGGCACATCAACTTCAC-3’
	Reverse	5’-GGGAGGGAGAGAGGAGAGAGG-3’		Reverse	5’-CCCTGTGTGTACTGGCCTTT-3’
Prdm16	Forward	5’-TGAGGAAGCATTTGAAGTTAAAG-3’	FASN	Forward	5'-TCGTCTATACCACTGCTTACTAC-3'
	Reverse	5’-GTTCTTAGCCTGCCTGTAC-3’		Reverse	5'-ACACCACCTGAACCTGAG-3'
PPARγ	Forward	5’-TGTGGACCTCTCCGTGATGG-3’	ACC1	Forward	5'-TGCCACCACCTTATCACTATGTA-3'
	Reverse	5’-GGTTCTACTTTGATCGCACTTTGG-3’		Reverse	5'-CCTGCCTGTCTCCATCCA-3'
Tbx-1	Forward	5’-GCGGAAGGAAGTGGTATT-3’	ACC2	Forward	5’-CCAACAGTAAGGTGGAAGCC-3’
	Reverse	5’-CTCTCTCGGTCGTCTACA-3’		Reverse	5’-CAGGGAGTTTCCTCTGCTGAC-3’
Tmem26	Forward	5’-GTCTCTACAACCTCCTGCTCTG-3’	CD36	Forward	5’-ATTCCCTTGGCAACCAACCA-3’
	Reverse	5’-TGTGCTATGCCGTTCTGTCTAC-3’		Reverse	5’-TACGTGGCCCGGTTCTACTA-3’
Cidea	Forward	5’-CCAAGGTCGGGTCAAGTCGTC-3’	LPL	Forward	5’-GGCTGACACTGGACAAACAAA-3’
	Reverse	5’-CGTAGTCCCTGGCGGTCTCC-3’		Reverse	5’-CCTGGGTTAGCCACCGTTTA-3’
β-actin	Forward	5’-GTTGGTTGGAGCAAACATC-3’	FATP5	Forward	5’-AGGACCAGCTGCATCCTTC-3’
	Reverse	5’-CTTATTTCATGGATACTTGGAATG-3’		Reverse	5’-TCTCCTACGCGTCGTACATTC-3’
HSL	Forward	5'-TGTGTCAGTGCCTATTCAG-3'	mt-DNA Loop1	Forward	5’-AATCTACCATCCTCCGTGAAACC-3’
	Reverse	5'-GAACAGCGAAGTGTCTCT-3'		Reverse	5’-TCAGTTTAGCTACCCCCAAGTTTAA-3’
ATGL	Forward	5'-GCTGTGGAATGAGGACATAGGA-3'	mt-DNA Loop2	Forward	5’-CCCTTCCCCATTTGGTCT-3’
	Reverse	5'-GCATAGTGAGTGGCTGGTGAA-3'		Reverse	5’-TGGTTTCACGGAGGATGG-3’
CPT1	Forward	5’-ACTCCGCTCGCTCATTCCG-3’	mt-DNA Loop3	Forward	5’-TCCTCCGTGAAACCAACAA-3’
	Reverse	5’-CACACCCACCACCACGATAA-3’		Reverse	5’-AGCGAGAAGAGGGGCATT-3’
MCAD	Forward	5’-GATCGCAATGGGTGCTTTTGATAGAA-3’	mt-ND4	Forward	5’-AACGGATCCACAGCCGTA-3’
	Reverse	5’-AGCTGATTGGCAATGTCTCCAGCAAA-3’		Reverse	5’-AGTCCTTCGGGCCATGATT-3’
HMGCS2	Forward	5’-ATACCACCAACGCCTGTTATGG-3’	CYTB	Forward	5’-GCTTTCCACTTCATCTTACCATTTA-3’
	Reverse	5’-CAATGTCACCACAGACCAG-3’		Reverse	5’-TGTTGGGTTGTTTGATCCTG-3’
PPARα	Forward	5’-TCGCTATCCAGGCAGAAG-3’	Tert	Forward	5’-CTAGCTCATGTGTCAAGACCCTCTT-3’
	Reverse	5’-ACCACAGACCAACCAAGT-3’		Reverse	5’-GCCAGCACGTTTCTCTCGTT-3’
Ckmt1	Forward	5’-TGAGGAGACCTATGAGGTATTTGC-3’	Ckmt2	Forward	5’-GCATGGTGGCTGGTGATGAG-3’
	Reverse	5’-TCATCAAAGTAGCCAGAACGGA-3’		Reverse	5’-AAACTGCCCGTGAGTAATCTT G-3’
Gamt	Forward	5’-GCAGCCACATAAGGTTGTTCC-3’	Gatm	Forward	5’-GACCTGGTCTTGTGCTCTCC-3’
	Reverse	5’-CTCTTCAGACAGCGGGTACG-3’		Reverse	5’-GGGATGACTGGTGTTGGAGG-3’
Slc6a8	Forward	5’-TGCATATCTCCAAGGTGGCAG-3’	Serca1	Forward	5’-TGTTTGTCCTATTTCGGGGTG-3’
	Reverse	5’-CTACAAACTGGCTGTCCAGA-3’		Reverse	5’-AATCCGCACAAGCAGGTCTTC-3’
Serca2a	Forward	5’-GCTCATTTTCCAGATCACACCG-3’	Serca2b	Forward	5’-ACCTTTGCCGCTCATTTTCCAG-3’
	Reverse	5’-GTTACTCCAGTATTGCGGGTTG-3’		Reverse	5’-AGGCTGCACACACTCTTTACC-3’
Serca3	Forward	5’-GGAGCAGTTTGAGGACCTCTT-3’	Fabp4	Forward	5’-AAG GTG AAG AGC ATC ATA ACC CT-3’
	Reverse	5’-GGCCACGAGAATTAGCATGATG-3’		Reverse	5’-TCA CGC CTT TCA TAA CAC ATT CC-3’
