# Supplementary material for: Mitochondrial stress-activated cGAS-STING pathway inhibits thermogenic program and contributes to overnutrition-induced obesity in mice
Source: Commun Biol. 2020 May 22;3:257. doi: 10.1038/s42003-020-0986-1 (PMC7244732; doi:10.1038/s42003-020-0986-1)
Supplement: Supplementary file 3 — Description of Additional Supplementary Files [file 42003_2020_986_MOESM3_ESM.pdf]

Description of Additional Supplementary Files

File Name: **Supplementary Data 1**
Description: source data
