# Supplementary material for: Mitochondrial stress-activated cGAS-STING pathway inhibits thermogenic program and contributes to overnutrition-induced obesity in mice
Source: Commun Biol. 2020 May 22;3:257. doi: 10.1038/s42003-020-0986-1 (PMC7244732; doi:10.1038/s42003-020-0986-1)
Supplement: Supplementary file 4 — Reporting Summary [file 42003_2020_986_MOESM4_ESM.pdf]

nature research

Corresponding author(s): COMMSBIO-19-1675-B Dr. Feng Liu, Dr. Juli Bai

Last updated by author(s): Apr 15, 2020

Reporting Summary

Nature Research wishes to improve the reproducibility of the work that we publish. This form provides structure for consistency and transparency in reporting. For further information on Nature Research policies, see Authors & Referees and the Editorial Policy Checklist.

Statistics

For all statistical analyses, confirm that the following items are present in the figure legend, table legend, main text, or Methods section.

n/a	Confirmed	
☐	☒	The exact sample size (*n*) for each experimental group/condition, given as a discrete number and unit of measurement
☐	☒	A statement on whether measurements were taken from distinct samples or whether the same sample was measured repeatedly
☐	☒	The statistical test(s) used AND whether they are one- or two-sided<br>*Only common tests should be described solely by name; describe more complex techniques in the Methods section.*
☒	☐	A description of all covariates tested
☒	☐	A description of any assumptions or corrections, such as tests of normality and adjustment for multiple comparisons
☐	☒	A full description of the statistical parameters including central tendency (e.g. means) or other basic estimates (e.g. regression coefficient) AND variation (e.g. standard deviation) or associated estimates of uncertainty (e.g. confidence intervals)
☐	☒	For null hypothesis testing, the test statistic (e.g. *F*, *t*, *r*) with confidence intervals, effect sizes, degrees of freedom and *P* value noted<br>*Give P values as exact values whenever suitable.*
☒	☐	For Bayesian analysis, information on the choice of priors and Markov chain Monte Carlo settings
☒	☐	For hierarchical and complex designs, identification of the appropriate level for tests and full reporting of outcomes
☒	☐	Estimates of effect sizes (e.g. Cohen's *d*, Pearson's *r*), indicating how they were calculated

Our web collection on statistics for biologists contains articles on many of the points above.

Software and code

Policy information about availability of computer code

Data collection	No software was used.
Data analysis	Data analysis was conducted using GraphPad Prism (Ver. 7.04), Microsoft Excel (Excel 2017).

For manuscripts utilizing custom algorithms or software that are central to the research but not yet described in published literature, software must be made available to editors/reviewers. We strongly encourage code deposition in a community repository (e.g. GitHub). See the Nature Research guidelines for submitting code & software for further information.

Data

Policy information about availability of data

All manuscripts must include a data availability statement. This statement should provide the following information, where applicable:

- Accession codes, unique identifiers, or web links for publicly available datasets
- A list of figures that have associated raw data
- A description of any restrictions on data availability

The authors declare that the data supporting the findings of this study are available within the article and from the corresponding author on reasonable request.

Field-specific reporting

Please select the one below that is the best fit for your research. If you are not sure, read the appropriate sections before making your selection.

☒ Life sciences ☐ Behavioural & social sciences ☐ Ecological, evolutionary & environmental sciences

For a reference copy of the document with all sections, see nature.com/documents/nr-reporting-summary-flat.pdf

October 2018

Life sciences study design

All studies must disclose on these points even when the disclosure is negative.

Sample size	Animal n number was indicated in each figures. Three independent replicates were performed for all cell studies.
Data exclusions	No data were excluded.
Replication	All attempts at replication were successful.
Randomization	not applicable
Blinding	not applicable

Reporting for specific materials, systems and methods

We require information from authors about some types of materials, experimental systems and methods used in many studies. Here, indicate whether each material, system or method listed is relevant to your study. If you are not sure if a list item applies to your research, read the appropriate section before selecting a response.

Materials & experimental systems

n/a	Involved in the study	
☐	☒	Antibodies
☐	☒	Eukaryotic cell lines
☒	☐	Palaeontology
☐	☒	Animals and other organisms
☒	☐	Human research participants
☒	☐	Clinical data

Methods

n/a	Involved in the study	
☒	☐	ChIP-seq
☒	☐	Flow cytometry
☒	☐	MRI-based neuroimaging

Antibodies

Antibodies used

1. Rabbit monoclonal anti-STING, Cell Signaling Technology, Cat# 13647;
2. Rabbit monoclonal anti-cGAS, Cell Signaling Technology, Cat# 31659
3. Rabbit monoclonal anti-Phospho-TBK1 (Ser172), Cell Signaling Technology, Cat# 5483
4. Rabbit polyclonal anti-TBK1, Cell Signaling Technology, Cat# 3013
5. Rabbit monoclonal anti-NF-κB p-p65, Cell Signaling Technology, Cat# 8242
6. Rabbit polyclonal anti-Phospho-NF-κB p-p65, Cell Signaling Technology, Cat# 3031
7. Rabbit monoclonal anti-TNFα, Cell Applications, Cat# CG1601
8. Rabbit monoclonal anti-Phospho-IRF3 (Ser396), Cell Signaling Technology, Cat# 4947
9. Rabbit monoclonal anti-IRF3, Cell Signaling Technology, Cat# 4302
10. Rabbit polyclonal anti-UCP1, Abcam, Cat# Ab23841
11. Rabbit monoclonal anti-C/EBPβ, Abcam, Cat# Ab32358
12. Rabbit polyclonal anti-PGC1α, EMD Millipore, Cat# ST1204
13. Rabbit polyclonal anti-Phospho-(Ser/Thr) PKA substrate, Cell Signaling Technology, Cat# 9621
14. Rabbit polyclonal anti-Phospho-HSL(Ser563), Cell Signaling Technology, Cat# 4139
15. Rabbit polyclonal anti-HSL, Cell Signaling Technology, Cat# 4107
16. Rabbit monoclonal anti PPARγ, Cell Signaling Technology, Cat# 2443
17. Rabbit polyclonal anti-Prdm16, Abcam, Cat# ab106410
18. Rabbit monoclonal anti-ATGL, Cell Signaling Technology, Cat# 2439
19. Rabbit monoclonal anti-Fasn, Cell Signaling Technology, Cat# 3180
20. Rabbit polyclonal anti-p-ACC (Ser79), Cell Signaling Technology, Cat# 3661
21. Rabbit polyclonal anti-ACC, Cell Signaling Technology, Cat# 3662
22. Rabbit polyclonal anti-ChREBP, Abcam, Cat #157153
23. Rabbit monoclonal anti-SCD1, Cell Signaling Technology, Cat# 2794
24. Mouse monoclonal anti-Complex IV, Molecular Probes, Cat# A6403
25. Rabbit polyclonal anti-Erp57, Abcam, Cat# Ab10287
26. Rabbit polyclonal anti-Lamin A, Bio Vision, Cat# 3267-100
27. Rabbit polyclonal anti-Actin, Cell Signaling Technology, Cat# 4967
28. Mouse momoclonal anti-β-Tubulin, Homemade
29. Mouse monoclonal anti-Myc-Tag, Homemade
30. Rabbit polyclonal anti-DsbA-L, Homemade, Also available in EMD Millipore, Cat# ABS1644

Validation

The validation of commercial antibodies used in this study were posted in manufacturer's website. The homemade antibodies were validated in our previous studies: Liu M, Zhou L, Xu A, Lam KS, Wetzel MD, Xiang R, Zhang J, Xin X, Dong LQ, Liu F. A

disulfide-bond A oxidoreductase-like protein (DsbA-L) regulates adiponectin multimerization.Proc Natl Acad Sci U S A. 2008 Nov 25;105(47):18302-7.

Eukaryotic cell lines

Policy information about cell lines

Cell line source(s)	The brown adipocytes was a gift by Dr. Jiandie Lin, University of Michigan. MEF and RAW264.7 cells were purchased from ATCC.
Authentication	The cell line was described in Wang GX, Zhao XY, Meng ZX, Kern M, Dietrich A, Chen Z, Cozacov Z, Zhou D, Okunade AL, Su X, Li S, Blüher M, Lin JD. The brown fat-enriched secreted factor Nrg4 preserves metabolic homeostasis through attenuation of hepatic lipogenesis. Nat Med. 2014 Dec;20(12):1436-1443.
Mycoplasma contamination	not contaminated
Commonly misidentified lines (See ICLAC register)	not applicable

Animals and other organisms

Policy information about studies involving animals; ARRIVE guidelines recommended for reporting animal research

Laboratory animals	Adult male (8+ weeks) DsbA-L fat-specific knockout and DsbA-L loxp mice, cGAS knockout mice (Jackson Laboratory, Stock No. 026554), STING deficient mice (also known as Goldenticket mice, Jackson Laboratory, Stock No. 017537) and C57BL/6J were utilized for experimental procedures.
Wild animals	This study did not involved wild animals.
Field-collected samples	The study did not involves samples collected from the field.
Ethics oversight	All animal experiment protocols were approved by the Institutional Animal Care and Use (IACUC) committee at University of Texas Health San Antonio and performed in accordance with the Guidelines for the Care and Use of Laboratory Animals of the National Institutes of Health.

Note that full information on the approval of the study protocol must also be provided in the manuscript.
